# let-7-repressesed Shc translation delays replicative senescence

**DOI:** 10.1111/acel.12176

**Published:** 2013-11-25

**Authors:** Fang Xu, Lijun Pang, Xiaoyu Cai, Xinwen Liu, Shuai Yuan, Xiuqin Fan, Bin Jiang, Xiaowei Zhang, Yali Dou, Myriam Gorospe, Wengong Wang

**Affiliations:** 1 Department of Biochemistry and Molecular Biology, Peking University Health Science Center38 Xueyuan Road, Beijing, 100191, China; 2 Department of Pathology and Biological Chemistry, University of MichiganMSI 5215A, 1301 Catherine Street, Ann Arbor, MI, 48105, USA; 3 Laboratory of Genetics, National Institute on Aging, National Institutes of Health251 Bayview Blvd., Baltimore, MD, 21224, USA

**Keywords:** cellular lifespan, let-7a, p66Shc, replicative senescence, translational regulation

## Abstract

The p66Shc adaptor protein is an important regulator of lifespan in mammals, but the mechanisms responsible are still unclear. Here, we show that expression of p66Shc, p52Shc, and p46Shc is regulated at the post-transcriptional level by the microRNA let-7a. The levels of let-7a correlated inversely with the levels of Shc proteins without affecting Shc mRNA levels. We identified ‘seedless’ let-7a interaction elements in the coding region of Shc mRNA; mutation of the ‘seedless’ interaction sites abolished the regulation of Shc by let-7a. Our results further revealed that repression of Shc expression by let-7a delays senescence of human diploid fibroblasts (HDFs). In sum, our findings link let-7a abundance to the expression of p66Shc, which in turn controls the replicative lifespan of HDFs.

## Introduction

Animal longevity is controlled by multiple molecular mechanisms, involving genes such as the silent information regulator SIRT1 (Bordone & Guarente, 2005), superoxide dismutases (SOD1 and SOD2; Fabrizio *et al*., 2003; Parker *et al*., 2004), p66Shc (Migliaccio *et al*., 1999; Pinton & Rizzuto, 2008), as well as insulin and insulin-like growth factor-1 (IGF-1; Yang *et al*., 2005). p66Shc is one of the members of the Shc family of proteins consisting of three isoforms (p66Shc, p52Shc, and p46Shc) that arise through alternative initiation of translation (Luzi *et al*., 2000). p52Shc and p46Shc were found in every cell type with invariant reciprocal relationship, while p66Shc expression varies in different cell type, suggesting that the function of p66Shc may be different from those of p52Shc/p46Shc (Migliaccio *et al*., 1997). Initially, all three isoforms of Shc were recognized as ‘adaptor’ proteins forming a complex with Grb2, an adaptor protein in the Ras signaling pathway. However, unlike p52Shc and p46Shc, p66Shc has little effect on the Ras signal pathway (Migliaccio *et al*., 1997). Instead, p66Shc has been described as a regulator for the longevity of mammals. p66Shc^−/−^ mice exhibited longer lifespan that was probably due to decreased cardiac diseases and reduced reactive oxygen species (ROS) production (Migliaccio *et al*., 1999; Napoli *et al*., 2003). However, the role of p66Shc in regulating other age-related processes has not been elucidated.

In response to oxidative stress, part of the cytosolic p66Shc translocates to the mitochondria and acts as an oxidoreductase. Phosphorylation at serine 36 by PKC is essential for its translocation, because a serine-phosphorylation defective mutant of p66Shc cannot restore the normal stress response in p66Shc^−/−^ cells (Migliaccio *et al*., 1999). On the other hand, elevation of p66Shc protein has been observed in primary human prostate tumors (Veeramani *et al*., 2005), in replicative senescence (Zhang *et al*. 2010), in middle-aged mice (Lebiedzinska *et al*., 2009), as well as in cells exposed to oxidative stress and UVC (Favetta *et al*., 2004). Regulation of p66Shc at transcriptional level by p53 and DNA methylation has been described (Trinei *et al*., 2002; Ventura *et al*., 2002). However, the regulation of p66Shc in replicative senescence remains largely unknown.

In this study, we show that microRNA let-7a regulates lifespan of human diploid fibroblasts by repressing the translation of p66Shc. We describe that let-7a interacts with ‘seedless’ sites located in the coding region (CR) of p66Shc mRNA, prevents the association p66Shc mRNA with the polysome, and enhances the recruitment of p66Shc mRNA into PBs, thereby repressing the translation of p66Shc. These studies reveal a novel microRNA-mediated mechanism linking p66Shc and cellular lifespan.

## Results

### let-7 represses the translation of p66Shc, p52Shc, and p46Shc

**Figure 1:**
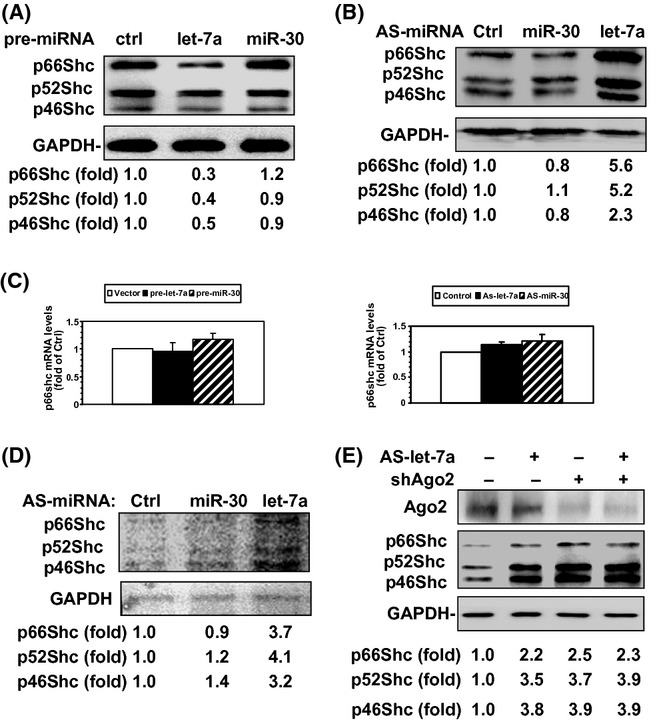
let-7a represses the translation of p66Shc. (A) IDH4 cells were transfected with a vector expressing the pre-miRNA of let-7a, miR-30, or an empty vector. Forty-eight hours later, cell lysate was prepared and subjected to Western blot analysis using anti-p66Shc or anti-GAPDH antibody. (B) IDH4 cells were transfected with a vector expressing AS-let-7a, AS-miR-30, or an empty vector. Forty-eight hour after transfection, Western blot analysis was performed using anti-p66Shc or anti-GAPDH antibody. (C) RNA samples described in panels (A,B) were subjected to RT-qPCR analysis to assess the mRNA levels of p66Shc. (D) Cells described in (A) (right) were incubated with L-[^35^S] methionine and L-[^35^S] cysteine (1 mCi mL^−1^) for 20 min, whereupon nascent p66Shc or GAPDH proteins were detected as previously described. (E) HeLa cells were either individually transfected with a vector expressing AS-let-7a (lane 2) or Ago2 (lane 3) shRNA or cotransfected with both vectors (lane 4). Forty-eight hour after transfection, Western blotting analysis was performed to assess the protein levels of Ago2, p66Shc, and GAPDH. All Western blotting data are representative from three independent experiments. The RT-qPCR data represent mean ± SD from three independent experiments.

This study was initiated from our findings that intervention of the let-7a levels altered the levels of p66Shc, p52Shc, and p46Shc proteins. Western blot analysis revealed that overexpression of let-7a by transfecting a vector that expressed pre-let-7a in IDH4 cells reduced p66Shc, p52Shc, and p46Shc proteins by ~50–70% (Fig. 1A), while knockdown of let-7a by transfecting a vector expressing let-7a antisense (AS-let-7a) increased Shc proteins by ~2.3- to 5.6-fold (Fig. 1B). IDH4 cells are derived from senescent IMR-90 cells, but through constitutive, dexamethasone (dex)-driven SV40 large T-antigen, IDH4 cells can proliferate in culture; upon dex removal from the medium, cells rapidly return to senescence (Wright *et al*., 1989). In contrast, neither knockdown nor overexpression of miR-30 substantially altered the expression of p66Shc, p52Shc, and p46Shc. To further address the mechanism by which let-7a regulates the expression of Shc proteins, the levels of p66Shc mRNA in cells described in Fig. 1(A,B) were analyzed by reverse-transcription (RT) followed by real-time, quantitative (q)PCR analysis. p52Shc and p46Shc were translated from the same transcript as p66Shc (p66Shc mRNA), by alterative initiation of translation (Fig. S1). The levels of p66Shc mRNA, which could potentially be used for synthesis of all Shc proteins, were not substantially altered by modulating let-7a or miR-30 abundance (Fig. 1C), suggesting that let-7a does not affect Shc expression at the level of mRNA turnover and instead may affect Shc translation. To test this hypothesis, IDH4 cells transiently expressing antisense let-7a or antisense miR-30 were incubated in medium containing L-[^35^S] methionine and L-[^35^S] cysteine for 20 min, cell lysates were then prepared and subjected to immunoprecipitation to analyze the level of nascent Shc proteins. As shown in Fig. 1(D), nascent Shc protein synthesis in AS-let-7a-expressing cells was ~3.2- to 4.1-fold higher than what was observed in control cells, while Shc translation in AS-miR-30-expressing cells was comparable with that measured in control cells. In control reactions, knockdown of let-7a or miR-30 did not influence the levels of nascent GAPDH. These results support the idea that let-7a represses the translation of p66Shc, p52Shc, and p46Shc.

To further investigate the repression of p66Shc, p52Shc, and p46Shc by let-7a, we examined the involvement of the Ago2-containing RNA/microRNA-induced silencing complex (RISC). HeLa cells were transfected with siRNA targeting Ago2 or let-7a, or co-transfected with both siRNAs, whereupon the levels of Shc proteins were assessed by Western blot analysis. As shown in Fig. 1(E), knockdown of Ago2 and let-7a increased the levels of Shc proteins by ~2.2–3.8- and ~2.5–3.9-fold, respectively, while simultaneous knockdown of Ago2 and let-7a did not show further effect of elevating Shc protein levels. These results support the view that let-7a represses the translation of p66Shc, p52Shc, and p46Shc in an Ago2/RISC-dependent manner.

We also assessed the expression of Shc proteins in cells transfecting with the siRNA or inhibitor of let-7a. As shown in the Fig. S2(A), transfection of IDH4 cells with let-7a siRNA, but not miR-30 siRNA, elevated the levels of Shc proteins. Transfection of IDH4 cells with an inhibitor of let-7a increased the levels of Shc proteins; transfection of cells with let-7b inhibitor moderately induced the Shc protein levels (Fig. S2B). In addition, transfection of cells with inhibitor of let-7c, let7d, miR-9, miR-22, or miR-30 did not alter the levels of Shc proteins (Fig. S2B,C). These results confirmed that let-7a specifically represses the translation of p66Shc, p52Shc, and p46Shc.

### let-7a represses the expression of Shc by associating with ‘seedless’ interaction elements in the CR of p66Shc mRNA

**Figure 2:**
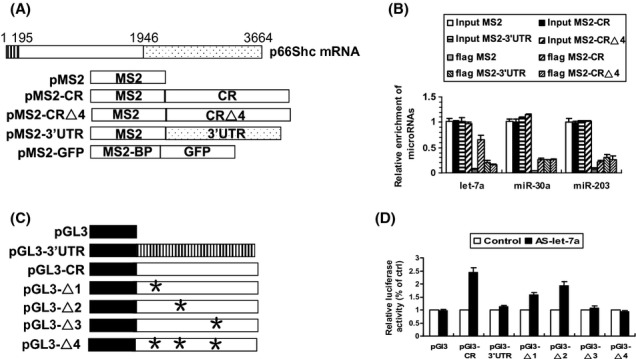
let-7 lowers p66Shc expression by interacting with the seedless recognition element (RE) in the coding region (CR) of p66Shc mRNA. (A) Schematic representation of the reporter vectors used for the RNP IP assays, the CRΔ4 derived from the CR fragment mutated all three ‘seedless’ REs. (B) Forty-eight hour after cotransfection of HeLa cells with pSL-GFP-MS2 and either pSL-MS2-CR, pSL-MS2-3′UTR, or pSL-MS2-CRΔ4, whole-cell lysates were prepared and subjected to IP assays using anti-GFP antibody (5 μg mL^−1^) or anti-IgG (5 μg mL^−1^), RNA were prepared from the IP materials and subjected to qPCR analysis to assess the levels of let-7a, miR-30a, and miR-203. The levels of microRNAs in pSL-MS2, pSL-MS2-CR, pSL-MS2-3′UTR, and pSL-MS2-CRΔ4 vector transfected cells are included (Input). Data represent the mean ± SE from three independent experiments. (C) Schematic representation of the pGL3-derived reporters used for reporter activity assays. (D) IDH4 cells were co-transfected with a vector expressing AS-let-7a or control antisense miRNA plus pGL3, pGL3-3′UTR, pGL3-CR, pGL3-Δ1, pGL3-Δ2, pGL3-Δ3, or pGL3-Δ4 reporter vector along with pRL-CMV control reporter. Forty-eight hour later, firefly luciferase activity was determined and normalized using renilla luciferase activity. Values represent the mean ± SD from three independent experiments.

By using the program RNA22, we identified three let-7a ‘seedless’ recognition elements (REs) – that is, sites of interaction lacking the conventional 7–8 nucleotide ‘seed’ that typically defines microRNA–mRNA interactions – in the CR of mRNA encoding p66Shc, p52Shc, and p46Shc (Fig. S1). To confirm the association of let-7a with the CR of Shc mRNA, HeLa cells were transfected with the reporter vectors pSL-MS2, pSL-MS2-CR, pSL-MS2-3′UTR or pSL-MS2-CRΔ4 together with the plasmid pSL-MS2-GFP-flag expressing the chimeric MS2-GFP-flag protein (Fig. 2A). The CRΔ4 was derived from the CR fragment mutating all three seedless REs of let-7a. This system allowed the immunoprecipitation of microRNAs interacting with the p66Shc mRNA fragments using anti-flag antibody (as the MS2-GFP-flag-MS2-CR, MS2-GFP-flag-MS2-CRΔ4, or MS2-GFP-flag-MS2-3′UTR complex; Zhang *et al*., 2012). The presence of microRNAs in the IP materials was assessed by RT-qPCR analysis. As shown in Fig. 2(B), the level of let-7a in the MS2-GFP-flag-MS2-CR complex was much higher than that in the MS2-GFP-flag-MS2-3′UTR or MS2-GFP–MS2-CRΔ4 complexes. As negative controls, the abundance of miR-30a or miR-203 in the MS2-GFP-flag-MS2-CR complex was comparable with that detected in MS2-GFP-flag-MS2-3′UTR and MS2-GFP-MS2-CRΔ4 complex. Therefore, let-7a interacts with the ‘seedless’ REs within the CR of Shc mRNA.

Next, we tested if the interaction of let-7a with the Shc CR was important for the regulation of Shc expression. We constructed pGL3-derived reporters bearing the p66Shc fragments 3′UTR, CR, or the CR fragment mutating the seedless interaction elements, as depicted in Figs S1 and 2(C). IDH4 cells were cotransfected with a vector expressing AS-let-7a or control antisense miRNA plus pGL3, pGL3-3′UTR, pGL3-CR, pGL3-Δ1, pGL3-Δ2, pGL3-Δ3, or pGL3-Δ4 reporter vector along with pRL-CMV control reporter, whereupon the firefly luciferase activity was analyzed. As shown in Fig. 2(D), knockdown of let-7a increased the luciferase activity from pGL3-CR by ~2.6-fold, but not from pGL3-3′UTR or pGL3-CRΔ4. In addition, knockdown of let-7a was less effective in elevating the luciferase activity of pGL3-CRΔ1 (~1.6-fold), pGL3-CRΔ2 (~1.9-fold), and pGL3-CRΔ3 (~1.1-fold). Together, interaction with the seedless REs of Shc mRNA was essential for let-7a to repress Shc translation.

The presence of an individual mRNA in polysomes and processing (P) bodies are indicators of the efficiency of translation of that mRNA. We therefore tested the presence of p66Shc mRNA in polysomes and p-bodies of cells with silenced let-7a. As shown in Fig. S3, knockdown of let-7a increased the presence of p66Shc mRNA in the polysome fraction while it reduced the presence of MS2-CR chimeric transcripts in P-bodies.

### Reduction of let-7a is accompanied by elevation of Shc proteins in replicative senescence

**Figure 3:**
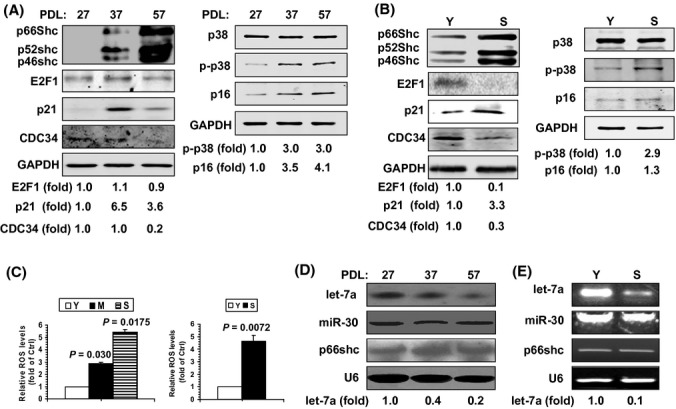
Expression of let-7a and p66Shc in replicative senescence. (A) Western blot analysis of p66Shc, p52Shc, p46Shc, E2F1, p21, CDC34, p38, p-p38, p16, and GAPDH in early- (~27 pdl), middle- (~37 pdl), and late-passage (~57 pdl) 2BS cells. (B) IDH4 cells were either cultured in the presence (Young, Y) or absence (Senescent, S) of Dexamethasone (Dex) for 5 days, whereupon total protein was prepared for Western blot analyses to assess the expression levels of p66Shc, p52Shc, p46Shc, E2F1, p21, CDC34, p38, p-p38, p16, and GAPDH. (C) Cellular ROS levels in cells described in (A,B) were analyzed. Data shown are the mean ± SD from three independent experiments and statistic significance was analyzed by Student’s *t*-test. (D,E) Total RNA prepared from cells described in (A,B) were used for Northern blot analysis (D) or semiquantitative PCR (E) to assess the levels of let-7a, miR-30, p66Shc, and U6. ROS, reactive oxygen species.

Next, we assessed the levels of Shc proteins in early-passage (Young, ~PDL 27), middle-passage (middle, ~PDL 37), and late-passage (Senescent, ~PDL 57) human diploid fibroblasts (2BS). As shown in Fig. 3(A), Shc proteins were almost undetectable in Young 2BS cells, but they increased in middle-passage cells and reached highest levels in senescent cells; similarly, Shc proteins were also higher in senescent IDH4 cells (Fig. 3B). The activity of p38MAPK (phospho-p38, p-p38) and the levels of p16 increased with cellular senescence (Fig. 3A,B). let-7 was found to suppress the expression of Ras, c-myc, E2F1, and CDC34, as well as to elevate the expression of p21. However, both Ras and c-myc were undetectable in either 2BS or IDH4 cells (data not shown); the levels of CDC34 decreased ~80% in senescent 2BS and ~70% in senescent IDH4 cells; the levels of p21 increased in middle-passage (~6.5-fold) and late-passage (~3.5-fold) 2BS cells as well as in senescent IDH4 cells (~3.3-fold; Fig. 3A,B). Although the levels of E2F1 remained unchanged in senescent 2BS cells, a remarkable reduction of E2F1 was observed in senescent IDH4 cells (~90%). In agreement with the findings that p66Shc is implicated in the production of intracellular ROS (Migliaccio *et al*., 1999; Napoli *et al*., 2003), ROS levels were significantly increased with the senescent process of both 2BS (*P* = 0.0175) and IDH4 cells (*P* = 0.0072; Fig. 3C).

Using the cells described in Fig. 3(A,B), the levels of let-7a, miR-30, U6, as well as p66Shc mRNA levels in 2BS and IDH4 cells progressing toward senescence were determined by Northern blot analysis (Fig. 3D) and by conventional RT-PCR analysis (Fig. 3E) respectively. In agreement with previous findings (Marasa *et al*., 2010), the levels of let-7a were reduced in middle-passage (~60%) and senescent (~80%) 2BS cells as well as in senescent IDH4 cells (~90%). In contrast, the levels of miR-30, U6, and p66Shc mRNA remained unchanged during senescence of 2BS (Fig. 3D) and IDH4 cells (Fig. 3E). These results suggest that the reduction of let-7a may contribute to the elevation of p66Shc, but not to the alterations of CDC34, E2F1, or p21 during replicative senescence.

### p66Shc reduces replicative lifespan of human diploid fibroblasts

**Figure 4:**
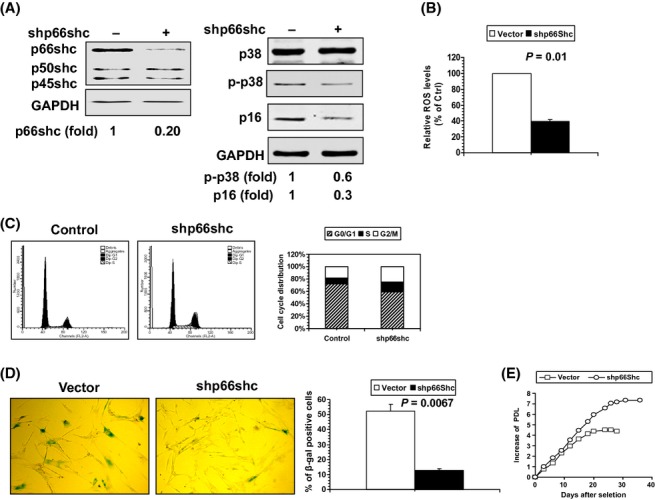
p66Shc negatively regulates cellular lifespan. (A) 2BS cells were stably transfected with a vector expressing p66Shc shRNA. The levels of p66Shc, p38, p-p38, p16, and GAPDH were assessed by Western blotting. (B) reactive oxygen species (ROS) levels were measured in cells treated as described in (A). (C,D) Cells described in (A) were used for FACS analysis (C) and SA-β-gal analysis (D). The results of SA-β-gal and ROS analysis are shown as the means ± SD and analyzed for statistical significance by Student’s *t*-test. (E) After selection, 1 × 10^5^ of cells described in (A) were cultured further, the cell numbers then were counted at times indicated, and the increase in PDLs is represented.

To address the role of p66Shc on cellular lifespan, p66Shc expression was silenced in 2BS cells by stably transfecting a vector that expressed p66Shc shRNA. This shRNA targets p66Shc, but not p52Shc or p46Shc (Data S1). As shown in Fig. 4(A), knockdown of p66Shc reduced the protein level of p66Shc (by ~80%), and p16 (by ~70%), as well as the levels of phopho-p38 (p-p38, by ~40%). In contrast, the levels of proteins p52Shc, p46Shc, and GAPDH remained unchanged. As a result, knockdown of p66Shc reduced intracellular ROS levels (*P* = 0.01; Fig. 4B), increased the S-phase compartment, reduced the G1 compartment (Fig. 4C), and lowered SA β-gal activity (*P* = 0.0067; Fig. 4D). After selection, the cells transfected with an empty vector essentially stopped dividing by day 20 and increased ~4.4 PDL, while the cells with silenced p66Shc stopped proliferating around day 30 and increased ~7.3 PDL (Fig. 4E). To further confirm the role of Shc proteins in replicative lifespan, 2BS cells were stably transfected with vectors expressing p66Shc, p52Shc, or p46Shc. As shown in Fig. S4(A), overexpression of p66Shc elevated the levels of proteins p66Shc (~3.3 fold), p52Shc (~3.5-fold), p46Shc (~3.0-fold), and p16 (~3.6-fold), as well as the levels of phospho-p38 (~2.8-fold). As anticipated, cells overexpressing p66Shc exhibited increased ROS levels (*P* = 0.0093; Fig. S4B), reduced S-phase compartment, increased G1 compartment (Fig. S4C), increased SA β-gal activity (*P* = 0.0003; Fig. S4D), and shortened replicative lifespan (Fig. S4E). The activity of p38 (p-p38) was also elevated in cells overexpressing p52Shc or p46Shc (Figs S5A and S6A). However, overexpression of p52Shc or p46Shc did not significantly alter the levels of cellular ROS, cell growth, as well as the replicative senescence and lifespan (Figs S5B–E and S6B–E). These results suggest that p66Shc acts as a negative regulator of the cellular lifespan, while p52Shc and p46Shc do not.

### let-7a extends cellular lifespan by repressing p66Shc expression

**Figure 5:**
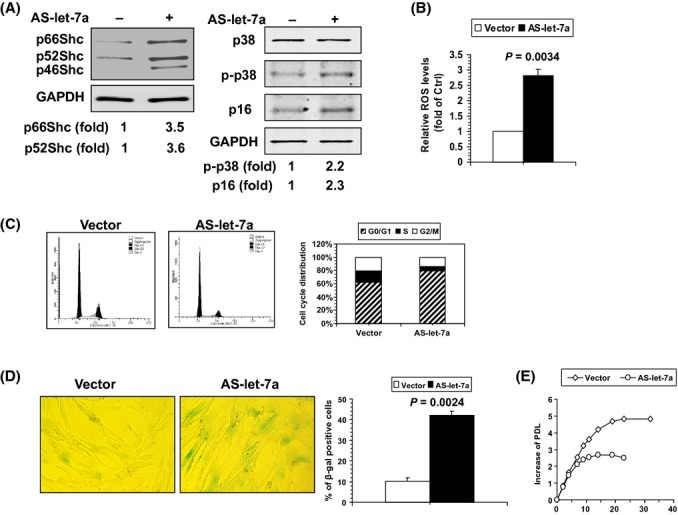
Reduction in let-7a shortens cellular lifespan. (A) 2BS cells were stably transfected with a vector expressing pre-let-7a. The levels of p66Shc and GAPDH were assessed by Western blotting. (B) ROS levels were measured in cells treated as described in (A). (C,D) Cells described in (A) were used for FACS analysis (C) and SA-β-gal analysis (D). The results of SA-β-gal and ROS analysis are represented as the means ± SD and analyzed for statistical significance by Student’s *t*-test. (D) After selection, 1 × 10^5^ of cells described in (A) were cultured further, the cell numbers then were counted at times indicated and the increase in PDLs is represented.

**Figure 6:**
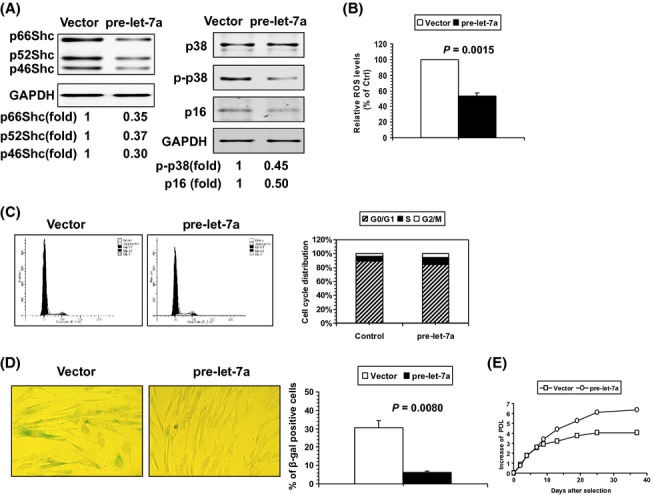
Overexpression of let-7a extends cellular lifespan. (A) 2BS cells were stably transfected with a vector expressing pre-let-7a. The levels of p66Shc, p38, p-p38, p16, and GAPDH were assessed by Western blotting. (B) reactive oxygen species (ROS) levels were measured in cells treated as described in (A). (C,D) Cells treated as described in (A) were used for FACS analysis (C) and SA-β-gal analysis (D). The results of SA-β-gal and ROS analysis were represented as mean ± SD and analyzed for statistical significance by Student’s *t*-test. (E) After selection, 1 × 10^5^ of cells described in (A) were cultured further, the cell numbers then were counted at times indicated and the increase in PDLs represented.

To address the impact of let-7a-p66Shc regulatory process upon replicative senescence, let-7a function in 2BS cells was either repressed or enhanced by stably transfecting cells with a vector expressing AS-let-7a or the pre-let7a, respectively. As shown in Fig. 5, cells expressing AS-let-7a increased the levels of p66Shc (~3.5-fold), p52Shc (~3.5-fold), p46Shc (undetectable in control cells, but increased by AS-let-7a), p16 (~2.3-fold), and phspho-p38 (~2.2-fold; Fig. 5A), increased ROS levels (*P* = 0.0034; Fig. 5B), reduced the S-phase compartment, increased the G1 compartment (Fig. 5C), and increased SA β-gal activity (*P* = 0.0024; Fig. 5D). After selection, AS-let-7a cells stopped proliferating around day 15, and increased ~2.7 PDL, while control cells stopped growing around day 25 and increased ~4.8 PDL (Fig. 5E). In contrast, cells expressing pre-let-7a exhibited reduced levels of Shc proteins (~65–70%), p16 (~50%), and phospho-p38 (~55%; Fig. 6A), reduced ROS levels (*P* = 0.0015; Fig. 6B), increased the S-phase cells and reduced G1-phase and SA-β-gal-positive cells (*P* = 0.0080; Fig. 6C,D). Cells expressing pre-let-7a continued to grow at day 37 after selection, with an increase in PDL ~6.4, while control cells stopped growing around day 25 after selection, with an increase in ~4.0 PDL (Fig. 6E). In addition, 2BS cells expressing a flag-tagged, frame-shifted p66Shc CR diminished the effect of let-7a in repressing p66Shc, p52Shc, p46Shc, p16, and the activation of p38, reducing ROS levels, promoting cell growth, delaying replicative senescence, and extending cell lifespan (Fig. S7). However, expression of the flag-p66Shc CRΔ4 was modest in rescuing the effect of expression of pre-let-7a (Fig. S7). Together, let-7a promoted cell proliferation, inhibited replicative senescence, and extended cell lifespan by repressing the production of p66Shc.

## Discussion

The findings presented in this study support the view that p66Shc negatively regulates the lifespan of human diploid fibroblasts (Fig. 4), in keeping with the finding that p66Shc deletion extends longevity in mammals (Migliaccio *et al*., 1999). We have found that the elevation of Shc proteins in replicative senescence is regulated at post-transcriptional levels by let-7a (Figs 6 and S2). By repressing the translation of Shc genes, let-7a extends the lifespan of human diploid fibroblasts (Figs 1, 5 and 6). Although the specific mechanisms by which let-7a represses the translation of Shc proteins are not fully understood, our data suggest that the association of let-7a with the seedless interaction elements located in the CR of Shc mRNA (Fig. 2C) lowers the presence of Shc mRNA in polysomes and enhances the recruitment of Shc mRNA into P-bodies (Fig. S3). The effect of let-7a was specific, as inhibition of let7c, let-7d, miR-9, miR-22, and miR-30 did not affect the levels of Shc proteins, while inhibition of let-7b moderately increased the levels of Shc proteins (Fig. S2). The let-7a-Shc regulatory process contributes at least in part to the elevation of Shc proteins in senescent cells, because expression of the Shc CR fragment antagonizes the effect of let-7a in regulating either Shc expression or cellular lifespan (Fig. S7). Given that the expression of Shc proteins in senescent cells is robustly elevated (Fig. 3) when let-7a levels decline, the let-7a-Shc regulatory paradigm may be in part responsible for the elevation of Shc proteins in replicative senescence.

P66Shc, p52Shc, and p46Shc are adaptors for the Ras-MAPK signaling pathway. In 2BS cells, p38 is activated by overexpression of p66Shc, p52Shc, or p46Shc, or by knockdown of let-7a, while p38 is inhibited by p66Shc knockdown or by let-7a overexpression (Figs 4A, 5A, 6A, S4A, S5A and S6A). Although the effects were most pronounced in the presence of active p66Shc, activation of p52Shc and p46Shc also modestly elevated ROS levels and elevated the senescence-associated β-galactosidase activity (Figs S5B and S6B). Given that overexpression of p66Shc and that let-7a lowered the levels of all three Shc isoforms (Figs S4 and 1), it is possible that p52Shc and p46Shc also regulate replicative senescence. In addition, other let-7-regulated mRNAs may also encode proteins involved in preventing senescence. Further work is needed to identify the complete set of effectors through which of let-7a impacts upon the cellular replicative lifespan.

MicroRNA-mediated gene regulation typically occurs through the interaction of the microRNA with the 3′UTR of the target mRNA (Bartel, 2009). However, analysis using general bioinformatics tools (Targetscan, miRanda, and microTv3.0), the p66Shc mRNA did not reveal any let-7 seed matches. Instead, our results indicate that seedless interaction elements in the CR of p66Shc mediate the association between let-7a and p66Shc mRNA and elicit the effects of let-7a (Fig. 2). MicroRNAs influence the translation or turnover of mRNAs as part of a larger molecular complex (the RISC) that includes Ago2 (Pratt & MacRae, 2009; Czech & Hannon, 2011); as anticipated, Ago2/RISC was involved in the let-7a-mediated regulation of Shc, because knockdown of Ago2 increased the levels of Shc proteins and diminished the effect of let-7 knockdown in elevating Shc expression (Fig. 1E).

p66Shc was described as a positive regulator of the proliferation of human prostate cancer cells (Veeramani *et al*., 2005). In human diploid fibroblasts, p66Shc may not promote cell growth, because elevated p66Shc levels have been observed in senescent human diploid fibroblasts (Fig. 3A) and in oxidative stress-induced senescence (Favetta *et al*., 2004). Indeed, evidence obtained in the present study supports the view that p66Shc acts as a negative regulator for cell growth (Figs 4C and S4C). On the other hand, let-7a inhibits the proliferation of human glioblastoma (Lee *et al*., 2011), human nonsmall cell lung tumor (Johnson *et al*., 2007; Kumar *et al*., 2008; He *et al*., 2009), Burkitt lymphoma (Sampson *et al*., 2007), breast cancer cells (Yu *et al*., 2007), and primary fibroblasts (Legesse-Miller *et al*., 2009), through the repression of Ras, c-myc, E2F1, and CDC34 as well as elevation of p21. However, these regulatory processes by let-7a may not impact on replicative senescence, as Ras and c-myc proteins were undetectable 2BS cells (not shown), and the reduction of let-7a in senescent cells was not accompanied by elevation of E2F1 and CDC34 or reduction of p21 (Fig. 3A,B).

The fact that let-7 represses proliferation of tumor cells but promotes the growth of HDFs may reflect the view that cell senescence contributes to tumorigenesis (Rodier & Campisi, 2011). In general, genes highly expressed in senescent cells tend to show low expression levels in cancer, and vice versa. Therefore, strategies to inhibit the growth of tumor cells may also shorten the lifespan of normal cells by inducing senescence, while strategies to extend cellular lifespan may increase the risk of tumorigenesis. Targeting let-7a-p66Shc in cancer may avoid this apparent dilemma, as extending cellular lifespan by elevating let-7a or reducing p66Shc does not increase the risk of tumorigenesis.

## Experimental procedures

### Cell culture and transfection

Human IDH4 fibroblasts were generously provided by J. W. Shay and cultured in Dulbecco’s modified Eagle’s medium supplemented with 10% fetal bovine serum, 100 units mL^−1^ penicillin, 100 μg mL^−1^ streptomycin, and dexamethasone (Dex, 1 μg mL^−1^) for constitutive expression of SV40 large T-antigen to suppress senescence and stimulate proliferation (Chang *et al*., 2010). Early- [Proliferating, Young, ~27 population doublings (pdl)], middle- (37 pdl), and late-passage (Senescent, ~57 pdl) human diploid 2BS fibroblasts (National Institute of Biological Products, Beijing, China), and HeLa cells were cultured in Dulbecco’s modified Eagle’s medium (Invitrogen, Grand Island, NY, USA) supplemented with 10% fetal bovine serum, 100 units mL^−1^ penicillin, and 100 μg mL^−1^ streptomycin, at 37°C in 5% CO_2_. All plasmids were transfected using lipofectamine 2000 (Invitrogen) and collected 48–72 h after transfection for further analysis. To establish 2BS cells stably expressing pre-let-7, AS-let-7a, p66Shc, p52Shc, p46Shc, or p66Shc shRNA, cells (~25 pdl) were transfected by lipofectamine 2000, selected by the G418 reagent (300 μg mL^−1^; Invitrogen) for 3–4 weeks, and maintained in medium supplemented with 50 μg mL^−1^ G418.

### RNA isolation, Northern blot, and PCR analysis

Total cellular RNA was prepared using the RNeasy Mini Kit (Qiagen, Hilden, Germany). Northern blot analysis was performed as described by Jing *et al*. (2005). For reverse-transcription (RT) followed by real-time, quantitative (q)PCR or semiquantitative PCR analysis of p66Shc and GAPDH, primers were described in ‘Supporting Information’. The primers for qPCR or semiquantitative PCR analysis of let-7a, miR-30, and U6 were from Ambion (Austin, TX, USA).

### Constructs and reporter gene assays

All vectors were constructed as described in ‘Supporting Information’. For reporter gene assays, each of the pGL3-derived vectors was cotransfected with pRL-CMV vector by Lipofectamine 2000 (Invitrogen). Forty-eight hour after transfection, cell lysates were collected and the firefly and renilla luciferase activities were measured with a double luciferase assay system (Promega, Madison, WI, USA) following the manufacturer’s instructions. All firefly luciferase measurements were normalized to renilla luciferase measurements from the same sample.

### Western blot analysis

Western blot analysis was performed as described (Chang *et al*., 2010). Monoclonal anti-GAPDH, polyclonal anti-p66Shc was from BD Biosciences (San Jose, CA, USA), Monoclonal anti-E2F1, anti-p21, anti-CDC34, and anti-p38, as well as polyclonal anti-p-p38 and p16 were from Santa Cruz (Santa Cruz, CA, USA). After secondary antibody incubation, signals were detected by SuperSignal WestPico Chemiluminescent Substrate (Pierce, Rockford, IL, USA) following the manufacturer’s instruction and quantitated by densitometric analysis with ImageMaster VDS software (Amersham Biosciences, Freiburg, Germany).

### Analysis of nascent protein and RNP IP assays

Nascent protein analysis was performed as described in ‘Supporting Information’. For RNP IP assays, HeLa Cells were cotransfected with reporter pSL-MS2 or pSL-MS2-CR along with the pSL-flag-MS2-GFP; 48 h later, cell lysates then were prepared and subjected to immunoprecipitation assays by using monoclonal antiflag antibody (Sigma, St. Louis, MO, USA). The IP materials were washed twice with stringent buffer (100 mm Tris-HCI, pH 7.4, 500 mm LiCI, 0.1% Triton X-100, 1 mm DTT, 2 μg mL^−1^ leupeptin, 2 μg mL^−1^ aprotinin, 1 mm phenylmethylsulfonyl fluoride), and twice with the IP buffer. RNA was isolated from the IP materials and analyzed by RT-qPCR to assess the levels of microRNAs (Zhang *et al*., 2012).

## Supporting Information

**Fig. S1** Schematic representation depicting the coding region (CR) and mutants of Src family mRNA. **Fig. S2** let-7a specifically represses the expression of p66Shc. **Fig. S3** let-7a inhibits the loading of p66Shc onto the p bodies but promotes the presence of p66Shc in the polysome. **Fig. S4** Overexpression of p66Shc shortens the life span and accelerates the replicative senescence of HDFs. **Fig. S5** Overexpression of p52Shc does not influence replicative senescence and life span. **Fig. S6** Overexpression of p46Shc does not influence replicative senescence and life-span. **Fig. S7** Ectopic expression of p66Shc CR fragment rescues the effect of let-7a on repressing p66Shc expression and extending the cellular life span. **Data S1** Experimental procedures.
