# Supplementary material for: let-7-repressesed Shc translation delays replicative senescence
Source: Aging Cell. 2013 Nov 25;13(1):185–92. doi: 10.1111/acel.12176 (PMC3947057; doi:10.1111/acel.12176)
Supplement: Supplementary file 1 — Fig. S1 Schematic representation depicting the coding region (CR) and mutants of Src family mRNA. Fig. S2 let-7a specifically represses the expression of p66Shc. Fig. S3 let-7a inhibits the loading of p66Shc onto the p bodies but promotes the presence of p66Shc in the polysome. Fig. S4 Overexpression of p66Shc shortens the life span and accelerates the replicative senescence of HDFs. Fig. S5 Overexpression of p52Shc does not influence replicative senescence and life span. Fig. S6 Overexpression of p46Shc does not influence replicative senescence and life-span. Fig. S7 Ectopic expression of p66Shc CR fragment rescues the effect of let-7a on repressing p66Shc expression and extending the cellular life span. Data S1 Experimental procedures. [file acel0013-0185-sd1.doc]

**Supporting Information**

**let-7 represses the translation of Shc genes in replicarive senescence**

Fang Xu^1^§, Lijun Pang^1^§, Xinwen Liu^1^, Shuai Yuan^1^, Xiuqin Fan^1^, Bin Jiang^1^, Xiaowei Zhang^1^, Yali Dou^2^, Myriam Gorospe^3^, and Wengong Wang^1*^

**Supplementary Experimental Procedures**

**Primers for RT-qPCR and vector construction.** For reverse transcription (RT) followed by real-time, quantitative (q)PCR or semiquantitative PCR analysis, primers CACTACCCTGTGCTCCTTCTTC and CGCCTCCACTCAGCTTGTT for p66Shc mRNA and primers CGAGTCAACGGATTTGGTGGTAT and AGCCTTCTCCATGGTGAAGAC for GAPDH mRNA were used.

For the construction of vectors expressing pre-let-7a and miR-30, the pre-let-7a and miR-30a were amplified using primers CGCGGATCCGCGTGGGATGAGGTAGTAGGT and CCATCGATGGTAGGAAAGACAGTAGATT, and primers CGCGGATCCGCGGCCACTTGCCTATTT and CCATCGATGGGCCCTACTACGCTTTT by PCR and inserted into the pLoxhyTk-LT vector (generously provided by Dr. Cong Y.) between the CalI and BamHI sites. For the construction of vectors expressing AS-let-7a, AS-miR-30, and p66Shc shRNA, GATCCCCTGAGGTAGTAGGTTGTATATTCAAGAGATATACAACCTACTACCTCATTTTTA and AGCTTAAAAATGAGGTAGTAGGTTGTATATCTCTTGAATATACAACCTACTACCTCAGGG for AS-let-7a,

GATCCCCTGGAAGCTGTGAAGCCACATTCAAGAGATGTGGCTTCACAGCTTCCATTTTTA and

AGCTTAAAAATGGAAGCTGTGAAGCCACATCTCTTGAATGTGGCTTCACAGCTTCCAGGG for AS-miR-30, and

GATCCCCTGAGTCTCTGTCATCGCTGTTCAAGAGACAGCGATGACAGAGACTCATTTTTA and

AGCTTAAAAATGAGTCTCTGTCATCGCTGTCTCTTGAACAGCGATGACAGAGACTCAGGG for p66Shc shRNA were inserted into pLoxhyTk-LT psuper.retro vector between the Bgl II and Hind III sites. To construct the pGL3-derived vectors for reporter gene assays, the p66Shc mRNA fragments CR (coding region, positions 550-1902) and 3’UTR (positions 1926-3323), were amplified by RT-PCR using following primers, primers TCATCTAGAGCGCAGGACTCGGGTGGAAGG and TCATCTAGACCGCAGAGATGATGGGCAAGTGAT for CR, Primers TCATCTAGACCTGTGGAGCGGAAACTGTGA and TCATCTAGAGGGCCGGCTTGCTGTGGTGT for 3’UTR. The CR fragments mutating the seedless recognition elements [CR-Δ1, CR-Δ2, CR-Δ3, and CR-Δ4 (mutating all three ‘seedless’ sites), Fig. 2C, schematic] were prepared by overlapping PCR. The fragments then were inserted into the XbaI site of pGL3-Basic vector (Promega, Madison). For constructing the pSL-MS2-CR, pSL-MS2-3’UTR and pSL-MS2-CRΔ4, fragments CR, 3’UTR, and CRΔ4 of p66Shc were inserted into the pMS2 vector (6×MS2 repeats). To construct pcDNA 3.1 vector expressing p66Shc, p52Shc, and p46Shc, the cDNA of p66Shc, p52Shc, and p46Shc was amplified by PCR (primers CGGAATTCCGATGGATCTCCTGCCCCCC and GCTCTAGAGCTCACAGTTTCC GCTCCAC for p66Shc, primers CGGAATTCCGATGAACAAGCT GAGTGGAGG and GCTCTAGAGCTCACAGTTTCCGCTCCACAG for p52Shc, and primers CGGAATTCCGATGGGACCCGGGGTTTCCTAC and GCTCTAGAGCGTTAGGGAA TAGGGTGGAAAG for p46Shc) from the pGL3-CR vector and inserted between the EcoRI and XbaI sites of the pcDNA.3.1 vector. To construct the pcDNA-p66Shc CR and pcDNA-p66Shc CRΔ vectors which expressing the flag-tagged and frame-shifted CR or CRΔ fragment, the p66Shc CR or p66Shc CRΔ fragment (positions 671-1900) was amplified from the pGL3-CR or pGL3-CRΔ vector by PCR using primers GGAATTCCATGGGTTTCC TACTTGTTCGGT and GCTCTAGAGCTTAGCAGAGATGATGGGCAAGTG, and inserted between the EcoRI and XbaI sites of the 3×flag pcDNA.3.1 vector (Zhang et al., 2012).

**Analysis of nascent protein.**  One million cells were incubated with 1 mCi (1 Ci = 37 GBq) L-[^35^S]methionine and L-[^35^S]cysteine (Easy Tag EXPRESS, NEN/Perkin–Elmer) per 60-mm plate for 20 min, whereupon cells were lysed by using TSD lysis buffer (50 mM Tris, pH 7.5/1% SDS/5 mM DTT), and lysates were immunoprecipitated by using either polyclonal anti-p66Shc antibody (BD) or monoclonal anti-GAPDH antibody (Santa Cruz Biotechnologies), or IgG for 1 h at 4°C. After extensive washes in TNN buffer (50 mM Tris, pH 7.5/250 mM NaCl/5 mM EDTA/0.5% Nonidet P-40), immunoprecipitated material was resolved by 12% SDS-PAGE, transferred onto PVDF membranes, and visualized by using a PhosphorImager (Molecular Dynamics).

**Preparation of polysomal fractions**

A total of 20 million cells were incubated for 15 min with 100 mg/ml cycloheximide, and total lysates (500 μl) were layered onto a cushion of 30% sucrose in ice-cold buffer containing 20 mM HEPES (pH 7.4), 50 mM potassium acetate, 5 mM magnesium acetate, 1 mM dithiothreitol, 1 unit of RNasin per μl, 1 μg of leupeptin per ml, 1 μg of aprotinin per ml, and 0.5 mM phenylmethylsulfonyl fluoride. After centrifugation (Beckman SW40; 100,000 × *g* for 2 h, 4°C), RNA from the supernatant (nonpolysomal fraction) and the pellet (polysomal fraction) was prepared and used for RT-qPCR analysis.

**Immunofluorescence and confocal microscopy**

Forty eight h after transfection of plasmids, cells were fixed with 4% formaldehyde, permeabilized with 0.5% Triton X-100, blocked with 5% BSA, and incubated with primary antibodies recognizing RCK (Santa Cruz Biotechnology). Alexa 405- or TRITC-conjugated secondary antibodies (Invitrogen) were used to detect primary antibody–antigen complexes with different color combinations as needed. The images were visualized using FV1000-ASW Olympus Micro with FV1000-ASV 1.6 Viewer image processing software. The confocal microscopy images were acquired with 10×100×2 and merged using maximum intensity.

**Measurement of the intracellular ROS**

Intracellular ROS levels were measured using dichlorodihydrofluorescein diacetate (H2DCFDA, Invitrogen), as described (Stephen et al., 2007; Wang and Joseph, 1999). Briefly, 1×10^4^ cells were plated into 96-well plates. Twenty four h later, cells were washed by HBSS (Hanks' Balanced Salt Solution) for 3 times and incubated with the loading medium containing H2DCFDA (10 μM) at 37°C for 15 min. The density of fluorescence measured at excitation488 nm and emission 525 nm. The final results were corrected for variations in the cell numbers and expressed as percentage (for reduction of ROS) or fold (for elevation of ROS) of control.

**Supplementary Figures**

**
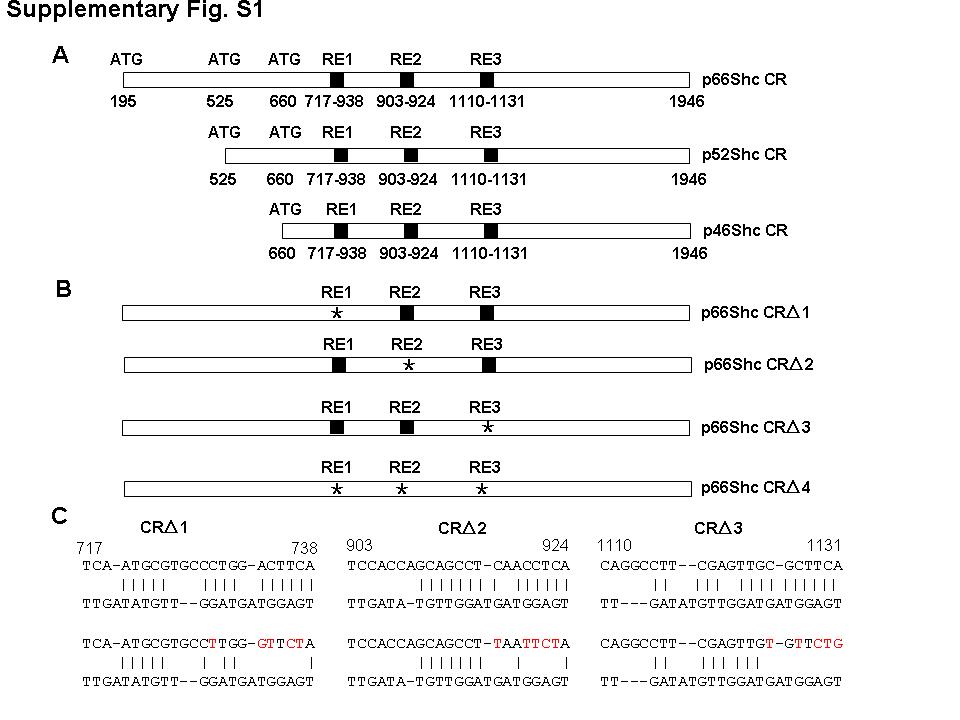
**

**Supplementary Fig. S1. Schematic representation depicting the coding region (CR) and mutants of Src family mRNA.**  **(A)** Schematic representation of the CR of p66Shc, p52Shc, and p46Shc. The positions of the translational initiation sites (ATG) and the recognition elements (**■,** RE1, RE2, and RE3) for miR-125b were indicated. **(B, C)** Schematic representation depicted the CRΔ1, CRΔ2, CRΔ3, and CRΔ4 mutants (B) and the mutation sites (C). The positions of each mutation was indicated as ‘**＊**’.

**
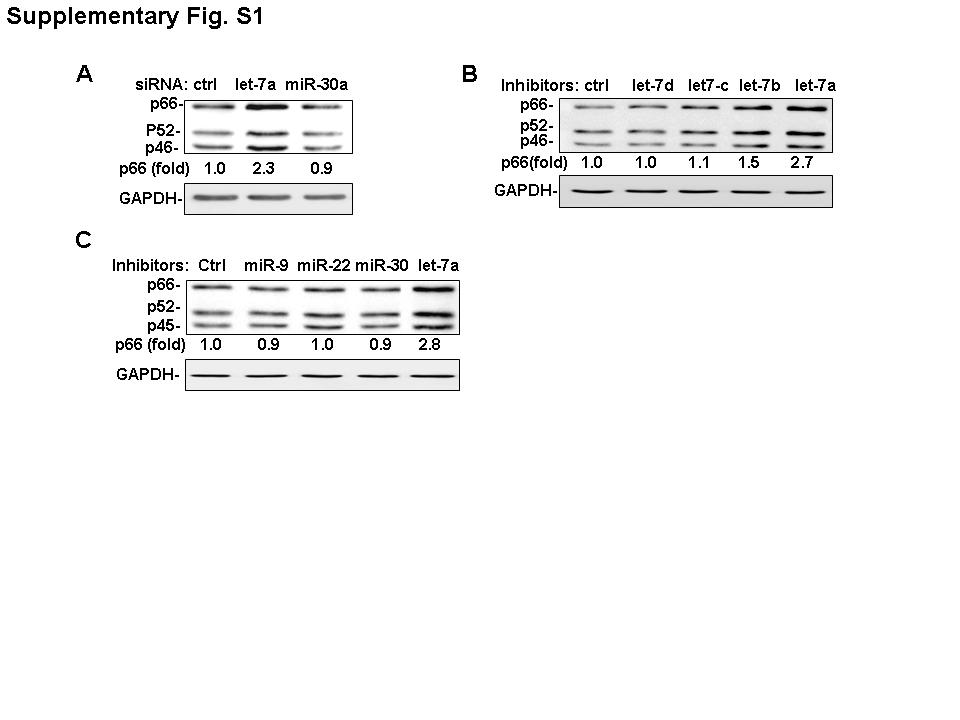
**

**Supplementary Fig. S2. let-7a specifically represses the expression of p66Shc. (A)** IDH4 cells were transfected with let7-a siRNA, miR-30 siRNA, or a control siRNA. Forty eight h later after transfection, cell lysate was prepared and subjected to Western blot to analyze the protein levels of p66Shc and GAPDH.  **(B)** IDH4 cells were transfected with inhibitors of let-7a, let-7b, let-7c, or let-7d. Forty eight h later, cell lysate was prepared and subjected to Western blot to analyze the protein levels of p66Shc and GAPDH. **(C)** IDH4 cells were transfected with inhibitor of let-7a, miR-9, miR-22, or miR-30. Forty eight h later, cell lysate was prepared and subjected to Western blot to analyze the levels of p66Shc and GAPDH.

**
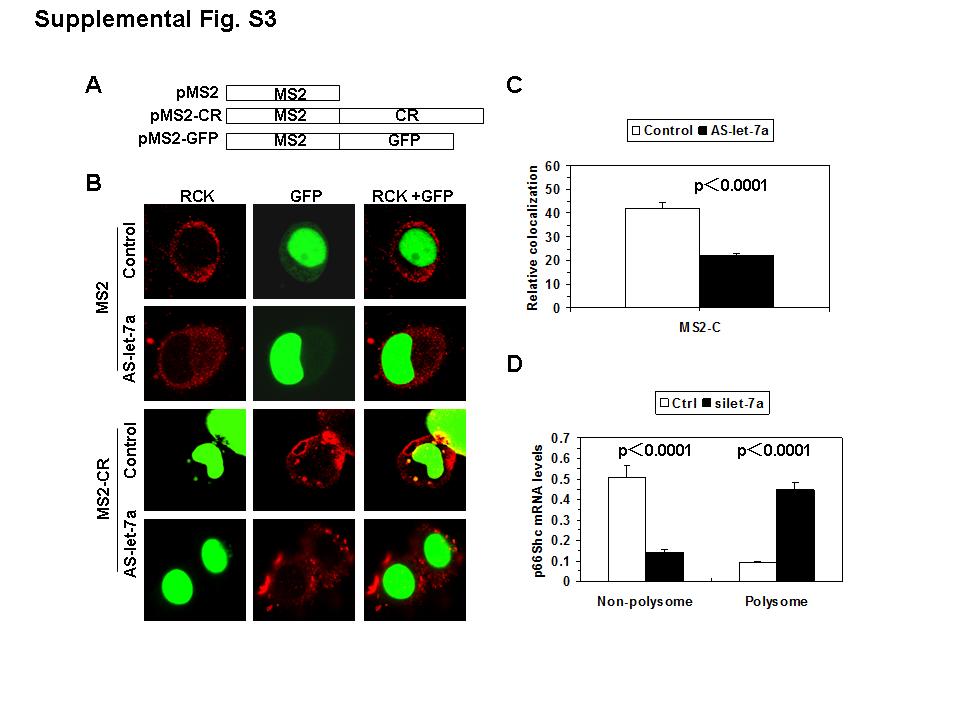
**

**Supplementary Fig. S3. let-7a inhibits the loading of p66Shc onto the p bodies but promotes the presence of p66Shc in the polysome. (A)** Schematic representation of the reporters used for P-body analysis. **(B)** 24 h after co-transfection of HeLa cells with pSL-GFP-MS2 and either pSL-MS2 or pSL-MS2-CR, cells were further transfected with vector expressing AS-let7a, and cultured for an additional 24 h. Using confocal microscopy, MS2 and MS2-CR were visualized using MS2-GFP (GFP, green fluorescence), and RCK signals (RCK, red immunofluorescence) were detected and colocalized (RCK + GFP, yellow). **(C)** The relative colocalization signals of GFP and RCK in (B) (percentage of (GFP + RCK) from total GFP signals in the cytoplasm) were calculated. Data of the relative colocalization signals of GFP and RCK are represented as the means ± SD from three independent experiments. The statistical significance of the relative colocalization of GFP and RCK was analysed by Student’s *t*-test. **(D)** HeLa cells were transfected with a siRNA targeting let-7a or a control siRNA; 48 h later, cell lysates were collected and used for isolation of the polysome fraction. The presence of p66Shc mRNA in the polysome and non-polysome fractions was assessed by real-time qPCR. Data represent the means ± SD from 3 independent experiments. The statistical significance was analyzed by Student’s *t* test.


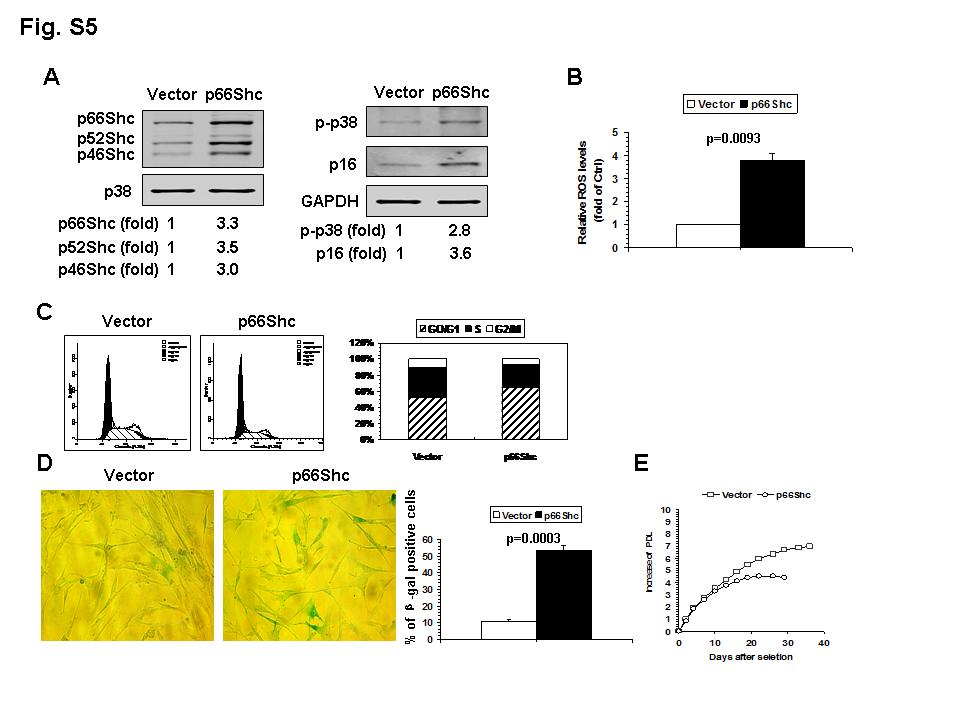


**Supplementary Fig. S4. Overexpression of p66Shc shortens the life span and accelerates the replicative senescence of HDFs. (A)** Human diploid fibroblasts (2BS) were transfected with a vector expressing p66Shc. Cells then were selected in G418 for 3 weeks, whereupon cell lysates were prepared and subjected to Western blot analysis to assess the protein levels of p66Shc, p-p38, p-38, p16, and GAPDH.  **(B, C, D)** Cells described in Fig.S4A were used for the analysis of relative ROS levels (B), FACS (C), and β-gal staining. (D). The data of ROS analysis and β-gal staining from three independent experiments were represented as mean±SD and analyzed for statistical significance by Student’s t test.  **(E)** After selection, 1×10^5^ of cells described in Fig. S4A were further cultured, the cell numbers then were counted at times indicated and the increase in PDLs is represented.


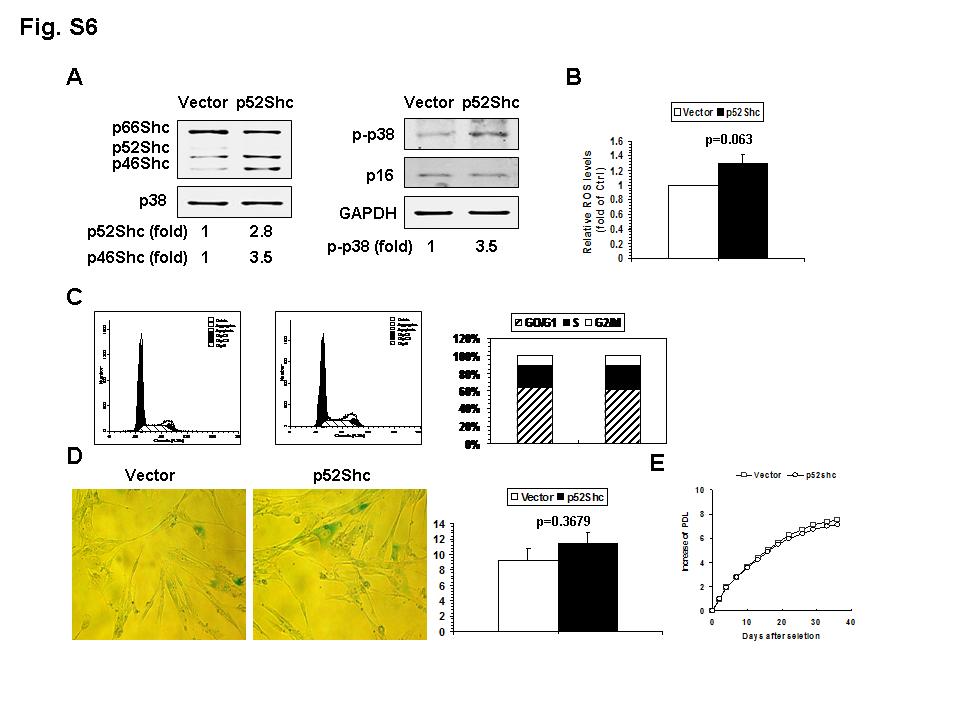


**Supplementary Fig. S5. Overexpression of p52Shc does not influence replicative senescence and life span.** **(A)** Human diploid fibroblasts (2BS) were transfected with a vector expressing p52Shc. Cells then were selected in G418 for 3 weeks, whereupon cell lysates were prepared and subjected to Western blot analysis to assess the protein levels of p52Shc, p-p38, p-38, p16, and GAPDH. The polyclonal anti-p66Shc antibody was used for Western blotting of p52Shc.  **(B, C, D)** Cells described in Fig.S5A were used for the analysis of relative ROS levels (B), FACS (C), and β-gal staining (D). The data of ROS analysis and β-gal staining from three independent experiments were represented as mean±SD and analyzed for statistical significance by Student’s t test. **(E)** After selection, 1×10^5^ of cells described in Fig. S5A were further cultured, the cell numbers then were counted at times indicated and the increase in PDLs is represented.


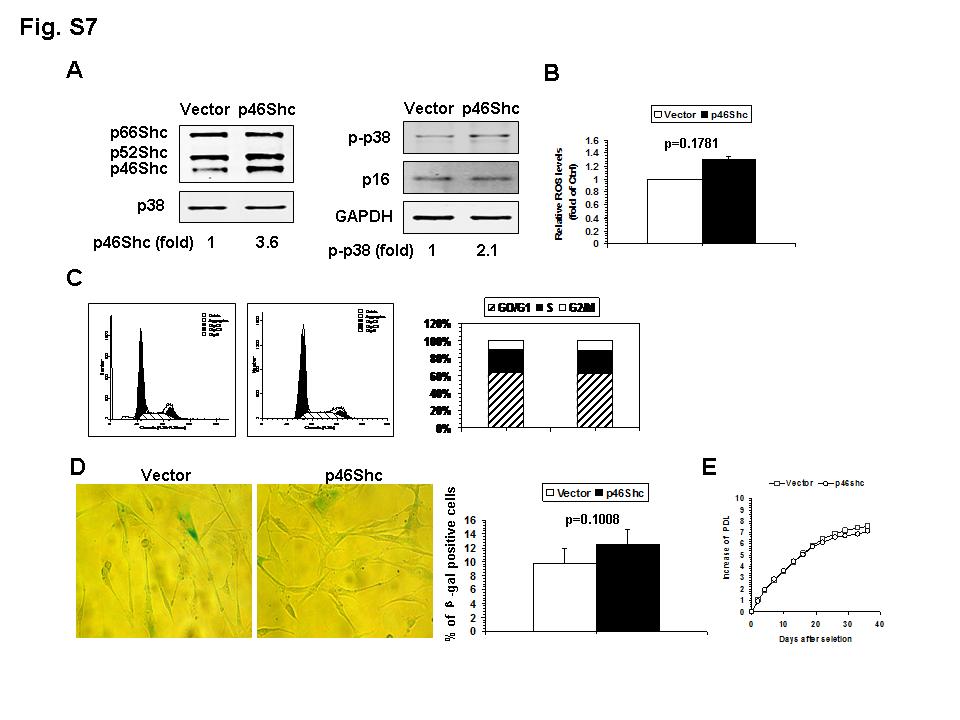


**Supplementary Fig. S6. Overexpression of p46Shc does not influence replicative senescence and life-span.** **(A)** Human diploid fibroblasts (2BS) were transfected with a vector expressing p46Shc. Cells then were selected in G418 for 3 weeks, whereupon cell lysates were prepared and subjected to Western blot analysis to assess the protein levels of p46Shc, p-p38, p-38, p16, and GAPDH. The polyclonal anti-p66Shc antibody was used for Western blotting of p46Shc.  **(B, C, D)** Cells described in Fig.S6A were used for the analysis of relative ROS levels (B), FACS (C), and β-gal staining (D), as described in Fig.S4B, S4C, and S4D. The data of ROS analysis and β-gal staining from three independent experiments were represented as mean±SD and analyzed for statistical significance by Student’s t test. **(E)** After selection, 1×10^5^ of cells described in Fig. S6A were further cultured, the cell numbers then were counted at times indicated and the increase in PDLs is represented.

**
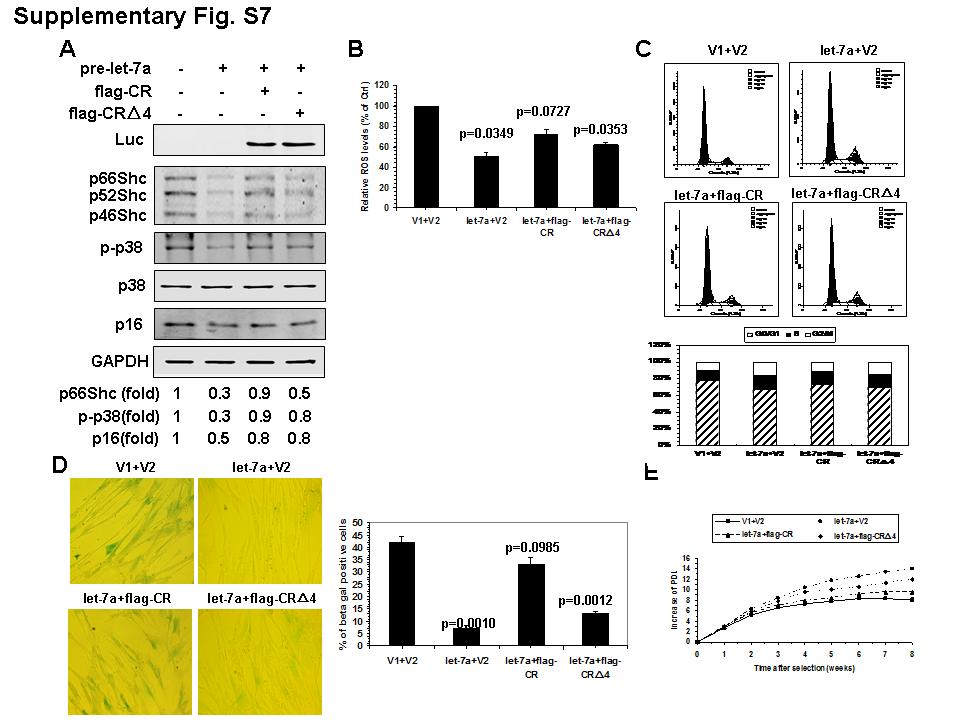
**

**Supplementary Fig. S7. Ectopic expression of p66Shc CR fragment rescues the effect of let-7a on repressing p66Shc expression and extending the cellular life span. (A)** Human diploid fibroblasts were co-transfected with a vector expressing pre-let-7a (H1-let-7a) and a vector expressing flag-tagged CR fragment of p66Shc (flag-CR) or the flag-tagged CR fragment mutating the let-7a recognition motifs (flag-CRΔ). Cells then were selected in G418 for 4 weeks, whereupon cell lysates were prepared and subjected to Western blot analysis to assess the protein levels of p66Shc, p-p38, p-38, p16, and GAPDH. **(B, C, D)** Cells described in Fig S7A were used for the analysis of relative ROS levels (B), FACS analysis (C) (V1, pLoxhyTk-LT empty vector; V2, pcDNA 3.1 empty vector.), and senescence associated β-gal staining (D). The data of ROS analysis andβ-gal staining from three independent experiments were represented as mean±SD and analyzed for statistical significance by Student’s t test. **(E)** After selection, 1×10^5^ of cells described in Fig. S7A were further cultured, the cell numbers then were counted at times indicated and the increase in PDLs is represented.

**References**

1. Stephen J. McNally, Ewen M. Harrison, James A. Ross, O. James Garden, Stephen J (2007) Wigmore Curcumin induces heme oxygenase 1 through generation of reactive oxygen species, p38 activation and phosphatase inhibition. *INTERNATIONAL JOURNAL OF MOLECULAR MEDICINE.* 19, 165-172.
2. Wang H, Joseph JA (1999) Quantifying cellular oxidative stress by dichlorofluorescein assay using microplate reader. *Free Radic Biol Med.* 27, 612-6.
